# Molecular Dynamics (MD) Simulation of GPR87-LPA Binding: Therapeutic Implications for Targeted Cancer Treatment

**DOI:** 10.2174/0118715206374428250403103159

**Published:** 2025-04-09

**Authors:** Mukta Rani, Amit Kumar Sharma, Anuradha Nischal, Sanjay Khattri, Ganesh Chandra Sahoo, Rajesh K. Singh

**Affiliations:** 1 Department of Bioinformatics, National Institute for Plant Biotechnology, Indian Council of Agricultural Research, Pusa Campus, New Delhi, 110012, India;; 2 Department of Pharmacology and Therapeutics, King George’s Medical University, Lucknow, 226003, U.P, India;; 3 Biomedical Informatics Centre, Institute of Medical Sciences, Indian Council of Medical Research, Agamkuan, Patna, 800007, India;; 4 Department of Physics, Faculty of Engineering, Teerthanker Mahaveer University, Moradabad, 244001, U.P., India;; 5 Department of Pharmaceutical Chemistry, Shivalik College of Pharmacy, Nangal, District Ropar, Punjab, 140126, India

**Keywords:** Cancer, GPCR, GPR87, lysophosphatidic acid, molecular dynamics, simulation, structure based drug targeting

## Abstract

**Background:**

GPR87 is an orphan G-protein-coupled receptor (GPCR) that represents a potential molecular target for developing novel drugs aimed at treating squamous cell carcinomas (SCCs) or adenocarcinomas of the lungs and bladder.

**Objectives:**

The present study aims to identify potential LPA analogues as inhibitors of the GPR87 protein through computational screening. To achieve this, the human GPR87 structure was modeled using template-based tools (Phyre2 and SWISS-MODEL), iterative threading (I-TASSER), and neural network-based de novo prediction (AlphaFold2). The modeled structures were then validated by assessing their quality against template structures using Verify-3D, ProSA, and ERRAT servers.

**Methods:**

We conducted a comprehensive structural and functional analysis of the target protein using various computational tools. Several computational techniques were employed to explore the structural and functional characteristics of the target, with LPA selected as the initial pharmacological candidate. A library of 2,605 LPA analogues was screened against orphan GPR87 through in-silico docking analysis to identify higher-affinity and more selective potential drugs.

**Results:**

Molecular dynamics (MD) simulations were performed to track structural changes and convergence during the simulations. Key metrics, including the root mean square fluctuation (RMSF) of Cα-atoms, radius of gyration, and RMSD of backbone atoms, were calculated for both the apo-form and the LPA-GPR87 complex structures. These studies on structure-based drug targeting could pave the way for the development of specific inhibitors for the treatment of squamous cell carcinomas.

**Conclusion:**

These findings may contribute to the design and development of new therapeutic compounds targeting GPR87 for the treatment of SCC.

## 1 INTRODUCTION

A significant proportion of human-derived G-protein Coupled Receptors (GPCRs) remain promising therapeutic targets and are believed to play a crucial role in drug discovery. It is estimated that the human genome contains over 900 GPCR genes [1]. GPCRs are a vast superfamily of membrane-bound signaling proteins that are highly valued for drug development in the pharmaceutical industry [2]. Approximately 50% of recently identified therapeutic targets-representing 26 out of the top 100 selling drugs are GPCRs [3]. GPCRs are involved in linking chronic inflammation to cancer, making them key targets in tumor-induced angiogenesis, metastasis, and the migration of cancer cells to target organs. As a result, GPCRs and their downstream signaling pathways present significant potential for the development of novel strategies in cancer diagnosis, prevention, and treatment [4].

Nearly 60% of currently marketed medications target GPCRs, underscoring their importance in therapeutic interventions. While new ligands for orphan GPCRs have been discovered, around 140 still remain uncharacterized [5, 6]. Identifying endogenous ligands for orphan GPCRs is essential for the design of drug-targeted receptors with specific physiological functions. Over the past two decades, reverse pharmacology has successfully deorphanized more than 300 GPCRs. These receptors act as signal transduction channels, connecting extracellular signals to various cellular responses [7, 8]. They serve as valuable models for studying receptor function and drug-ligand interactions, making them pivotal targets in drug development [9].

The orphan receptor GPR87, also known as GPR95, is a member of the P2Y12 receptor subgroup, which includes the P2Y12, P2Y13, P2Y14, CysLT1, and CysLT2 receptors [10]. Located on human chromosome 3q25 [11], the genes for these P2Y receptors are clustered in this region. Neoplastic cells in human Squamous Cell Carcinomas (SCCs) express human GPR87 (hGPR87) in various tissues, including the lung, cervical region, head and neck (larynx, pharynx, tonsils, and tongue), urinary bladder [12, 13], and placenta. GPR87 is a rhodopsin-type (class A) GPCR that functions as a pro-survival, p53-induced gene [14] and is implicated in skin cancers. Human GPR87 plays a critical role in intracellular signal transduction, normal ovarian development, and the progression of ovarian cancer [11]. It has also been shown to be overexpressed in lung adenocarcinoma, bladder carcinoma, and SCCs [13]. Recent studies have revealed that GPR87 plays a key role in modulating the expression of CD133 and contributes to the growth and metastasis of hepatocellular carcinoma (HCC) cells [15].

Two key reasons have emerged for targeting the human GPR87 protein. First, GPR87 is the only GPCR found to be overexpressed and associated with mutation levels in Squamous Cell Carcinomas (SCCs) of the lung. Second, it exhibits the highest fold increase in GPCR expression. GPR87 has also been identified as a novel target gene of p53, with overexpression observed in testicular cancer, potentially as a downstream effect of chemotherapy-induced p53 activation [16]. Other GPCRs also show promising roles in cancer research. For instance, GPR55 is overexpressed in pancreatic ductal adenocarcinoma and glioblastomas [17], while GPCR56 has recently been shown to be expressed in esophageal squamous cell carcinoma [18].

Knockdown experiments using small interfering RNA (siRNA) have demonstrated that hGPR87 plays a pivotal role in stimulating the proliferation of human tumor cell lines. Reducing the expression of hGPR87 through siRNA-mediated knockdown results in decreased cell proliferation, suggesting that hGPR87 is essential for the growth and survival of cancer cells. This underscores the potential of hGPR87 as a therapeutic target in cancer treatment, as its inhibition could disrupt tumor growth and enhance the efficacy of cancer therapies [16].

GPR87 survival is dependent on p53, and knocking down GPR87 for 72 hours leads to hyperactivation of p53, which is triggered by DNA damage. This suggests that GPR87 plays a role in regulating p53 activity, and its loss disrupts cellular stability, resulting in heightened p53 activation in response to DNA damage [14]. GPR87 may also be involved in the processes that drive tumor cell alterations, acting as a prosurvival factor and possibly mediating p53 in the prosurvival pathway [16]. The hGPR87 protein induces DNA damage in tumor cells *via* p53, positioning p53 as a downstream effector in the GPR87 signaling pathway.

Recent studies have shown that GPR87 is upregulated in the kidneys of individuals with Chronic Kidney Disease (CKD), suggesting its significant role in renal fibrosis through the promotion of glycolysis and mitochondrial damage [19]. As a member of the Lysophosphatidic Acid (LPA) receptor family, hGPR87 is thought to amplify signals to Akt, contributing to prosurvival activity. It shares a common ancestor with P2Y receptors [3]. LPA, a bioactive lipid with a single acyl chain, a phosphate group, and a glycerol backbone, activates six class A G-protein-coupled receptors, triggering a variety of cellular responses. Due to its involvement in cancer and fibrosis, LPA receptors are considered promising drug targets [4].

Lysophosphatidic acid activates the orphan GPR87, leading to an increase in intracellular Ca^2+^ levels in CHO cells engineered to express the GPR87-Gα16 fusion protein. This Ca^2+^ response is inhibited by the LPA receptor antagonist Ki16425, further supporting the role of GPR87 as an LPA receptor [20]. While X-ray crystallography has not yet provided structural data for the GPR87 protein, the modeled 3D structure of GPR87 remains a valuable tool for studying receptor function and designing pharmacological inhibitors.

This study presents a structural and functional analysis of the GPR87 protein using homology modeling to guide therapeutic screening through docking studies. As both a target of p53 and a prosurvival factor, GPR87 emerges as a promising candidate for cancer treatment. The research aims to identify potential inhibitors by screening LPA analogues through computational methods. While complete biochemical and structural data for GPR87 remain to be experimentally validated, this model serves as a valuable reference for designing inhibitors targeting squamous cell carcinomas. In doing so, the study contributes to the development of an anti-cancer drug discovery framework, supporting global efforts to combat cancer.

## 2 MATERIALS AND METHODS

### 2.1 Sequence Retrieval and Template Selection

The translated amino acid sequences of GPR87 in *Homo sapiens* (Accession Number: Q9BY21.1), consisting of 358 amino acids, were retrieved from the National Center for Biotechnology Information (NCBI). The homologous sequences of GPR87 were retrieved using a BLASTp [21] search against the Protein Data Bank (PDB). Among these sequences, the cryo-EM structure of the purinergic receptor P2Y12R in complex with 2MeSADP and Gi (PDB: 7XXI_A) [22] was selected as the best template for constructing the 3D structure of GPR87, based on its sequence identity (41.46%) and sequence similarity (62%). Sequences of P2Y receptors were categorized into two groups: Group I, which includes P2Y1, P2Y2, P2Y4, P2Y6, and P2Y11, and Group II, which includes P2Y12 and P2Y13. Additionally, pharmacologically related receptors such as CysLT1 and CysLT2 were also retrieved from NCBI. Multiple sequence alignment was performed using the ClustalW algorithm, and a cladogram was constructed using the TreeView program [23].

### 2.2 Modeling Studies and Validation

The modeled 3D structure of the hGPR87 protein was constructed using various tools and servers, including the Modeler module [24], Discovery Studio v2.5 [25], I-TASSER [26], Phyre2 [27], and SWISS-MODEL [28]. Additionally, neural network-based de novo modeling techniques, such as AlphaFold2 [29], were employed to achieve the best possible results by complementing each method.

Further, the coordinates obtained from modeled structures were visualized using the PyMOL program (http://www.pymol.org) [30]. The stereo-chemical quality, conformational correctness, and reliability of the modeled 3D structure of GPR87 were validated using the PROCHECK program [31]. Following structural validation, the ERRAT plot [32] was analyzed to identify structural errors in each residue. The modeling process was iteratively refined until the residues fell below the 95% cut-off value. Residues exceeding this threshold were subjected to loop modeling using the Modeler module.

Subsequently, the refined model was further validated using VERIFY-3D on the SAVES server [33]. ProSA was employed to assess the native protein folding energy [34], while the discrete optimized protein energy (DOPE) score was calculated using Discovery Studio v2.5 (DSv2.5). The model with the lowest DOPE score was considered the most accurate and was selected for further analysis [35].

### 2.3 Functional Analysis of hGPR87

The locations of the extracellular N-terminal domain and transmembrane domain in the GPR87 sequence were predicted using the PSI-PRED server [36]. To predict the transmembrane helices (TMs) in GPR87, we employed several computational methods. Thirteen different servers, including DAS, TMHMM, HMMTOP, TMpred, TopPred, SOSUI, SPLIT, PredictProtein (PHD), MEMSAT, WaveTM, HMM-TM, SMART, and Philius topology prediction [34-49], were used to predict and validate the transmembrane helical regions (TM regions) in the GPR87 protein. The binding sites of GPR87 were predicted using various servers, such as MetaPocket, LIGSITEs, PASS, Q-SiteFinder, SURFNET, and CASTp [50-55], to enhance the prediction accuracy and compare the results. MetaPocket [50], a suite of programs for identifying and characterizing protein binding sites and functional residues, was used to predict active sites in the GPR87 protein. Additionally, different binding cavities in GPR87 were predicted using Pocket-Finder, as discussed in a published research article [56]. The binding sites and their functional residues were identified and documented for further investigation.

### 2.4 Molecular Dynamics (MD) Simulations

Molecular dynamics (MD) simulations were performed on the modeled structure of GPR87 in an explicit solvent environment using the GROMACSv4.2 package, with the Gromos43a1 force field parameters applied for system setup and simulation [57]. The GPR87 model was solvated with a POPC lipid bilayer and 113,150 water molecules, modeled using the SPC/E water model. The system was placed within an octahedral box, with the edges positioned 0.9 nm away from the molecular periphery to ensure proper solvation and adequate space for MD simulations [58].

MD simulations were conducted in a periodic box with dimensions 96x96x96 nm and a spacing of 0.158 nm. To maintain system neutrality, Na^+^ ions (with a charge of +1.00) were added. The LINCS algorithm was employed to maintain the geometry of the molecules, and long-range electrostatic interactions were computed using the particle-mesh Ewald (PME) method. Electrostatic interactions within 9 Å were treated in direct space, while those beyond this distance were handled in Fourier space.

Position-restrained MD simulations were carried out under NPT conditions (constant number of particles, pressure, and temperature) for 10 ns, with trajectory snapshots taken every 0.5 ps. The final MD simulation consisted of 5,000,000 steps (10 ns) using the PME method for electrostatics under NPT conditions. GROMACS and VMD [59] were used to assess the quality of the simulation trajectories, and trajectory analysis graphs were generated with Xmgrace 4.1.2 (http://plasma-gate.weizmann.ac.il/Xmgr/). Visualizations were produced using PyMOL (http://www.pymol.org/).

### 2.5 Docking with Lysophosphatidic Acid (LPA) Analogs

GPR87, an orphan receptor, belongs to the P2Y and P2Y-related receptor family. Exploring novel ligands for P2Y and P2Y-related receptors to activate GPR87 could provide valuable insights for future research. Previous studies have shown that Lysophosphatidic Acid (LPA) can activate GPR87, indicating that GPR87 functions not as a nucleotide receptor but as an LPA receptor [6]. Elevated LPA levels have been detected in the plasma and ascitic fluid of ovarian cancer patients [60], suggesting that LPA plays a significant role in the pathology of various human cancers. Since GPR87 is considered part of the LPA receptor family, which is activated by lysophosphatidic acid, the characterization of LPA binding to GPR87 and its analogs was investigated through molecular docking and dynamics studies. Furthermore, several anti-cancer agents and their anti-proliferative activities were assessed against various cancer cell lines, providing insights into their protein-ligand interaction profiles [61]. For the docking studies, the FlexX program [62, 63] was utilized to dock the target protein with a virtual compound library of 2,605 LPA analogs, sourced from the PubChem compound database.

#### 2.5.1 FlexX Docking

Drug and ligand binding sites were simulated using the LeadIT software (BioSolve IT, GmbH). FlexX version 2.1.1 was employed as part of the analysis scheme, utilizing a variant of the SCORE1 scoring function, which was developed by Hans-Joachim Boehm for the *de novo* enzyme inhibitor design package LUDI [64]. This scoring function serves as a relevant consensus method, combining contributions from both FlexX and PLP scoring functions to improve the prediction of receptor-ligand binding energies. The composite scoring function is also used to rank the generated complex structures. The highest-ranked complex, determined in the rescoring step, is typically considered the final induced fit for the docked structure.

For refined pose rescoring, the consensus scoring function integrates the FlexX and PLP scoring functions with a molecular dynamics force field interaction energy derived from the minimized structural complex. Theoretically, the receptor-ligand interaction energy is defined as:

E_interact_ = [E_int complex_- (E_int receptor_+E_int ligand_)] + [E_sol complex_- (E_sol receptor_+E_sol ligand_)]

Where E_int_ and E_sol_ refer to the internal energy and solvation energy of the respective components, respectively.

In this equation, the standard parameters of FlexX were used for iterative growing and subsequent scoring of docked poses. The receptor description files for hGPR87 were automatically generated with coordinates in FlexX, resulting in 100 docked solutions. A consensus scoring function, combining two score values with equal weights, was then applied to select the top 20 ranked docked poses along with their corresponding binding site geometries. The different scoring conditions were combined in the consensus function using a scaling method, where the score of each model was scaled between 0.0 and 1.0 for each scoring function.

Further, the consensus score is defined as:

S_consensus_ = (W_PLP_ × S_PLP_ + W_FlexX_ × S_FlexX_ + W_interact_ × S_interact_) ⁄ (W_PLP_ + W_FlexX_ + W_interact_)

Where S consensus represents the consensus score and S_PLP_ is the normalized PLP score. S_FlexX_ is the normalized FlexX score, and S_interact_ is the normalized interaction energy. Using an empirical Monte Carlo optimization benchmark set of self-docking experiments, the following values were determined: w_PLP_ =5.0, w_FlexX_ = 1.0, and w_interact_ = 0.5.

In FlexX, lengths must be given in Å and energies in kJ/mol. For validation, we had previously used docking studies with anti-leishmanial compounds and interaction studies with other human GPCRs, such as EMR2, GAPDH protein in *Leishmania*, and the S-protein in SARS-CoV-2, which have been reported [65-67]. The apo-form and LPA analogues, which exhibited the highest binding affinity with the GPR87 protein (LPA-GPR87 complex structures), were further validated through MD simulations using the method described in section 2.4.

## 3 RESULTS AND DISCUSSION

### 3.1 Sequence Alignment

Multiple sequence alignments of the GPR87 protein with two groups of P2Y receptors were performed. Group I included P2Y1, P2Y2, P2Y4, P2Y6, and P2Y11, while Group II consisted of P2Y12 and P2Y13. Additionally, pharmacologically related receptors, such as CysLT1 and CysLT2, were included in the alignment. A distance matrix was calculated using ClustalW. The high sequence similarity between GPR87 and the P2Y, CysLT1, and CysLT2 receptors suggests a potential functional or structural relationship. Specifically, GPR87 in *Homo sapiens* shares 89% sequence similarity with the P2Y, CysLT1, and CysLT2 receptors in *Mus musculus*.

This information suggests that GPR87 in *Homo sapiens* and *Mus musculus* shares significant sequence similarity with certain P2Y receptors, as well as pharmacologically related receptors. Further studies may be warranted to explore the functional implications of these sequence similarities and their potential impact on receptor behavior or interaction in physiological processes.

### 3.2 Model Analysis and Structure Validation

Due to the lack of crystal structure information for the human GPR87 protein, a BLAST search was conducted against the PDB database to identify the best template for homology modeling. The 3D structure of the orphan GPR87 protein was constructed using the I-TASSER server, with the cryo-EM structure of the purinergic receptor P2Y12R in complex with 2MeSADP and Gi (PDB: 7XXI|A) serving as the template, which shares 44% sequence identity and 53% sequence similarity.

Four different homology modeling tools were used to construct the 3D structure of GPR87, and the modeled structure was validated using ProSA, ERRAT servers, and the HPM-score [68], as listed in Table **1**. When compared with the template, the selected homology model of GPR87 exhibited a ProSA score of -2.26, which was closest to that of the template (PDB ID: 7XXI), with a ProSA score of -2.18.

This model was generated using AlphaFold2 (Table **1**). When comparing the validation scores of the four modeled structures, it is evident that the 3D structure produced by the neural network-based de novo modeling technique, AlphaFold2, is the most suitable model for further analysis. The 3D structure of GPR87 was superimposed with the template (PDB ID: 7XXI_A), as shown in Fig. (**1a**). This study validated the 3D structure of GPR87 using a Ramachandran plot, which analyzed the Φ/Ψ backbone torsion angles for each residue, and further evaluated the model using the PROCHECK tool.

**Fig. (1):**
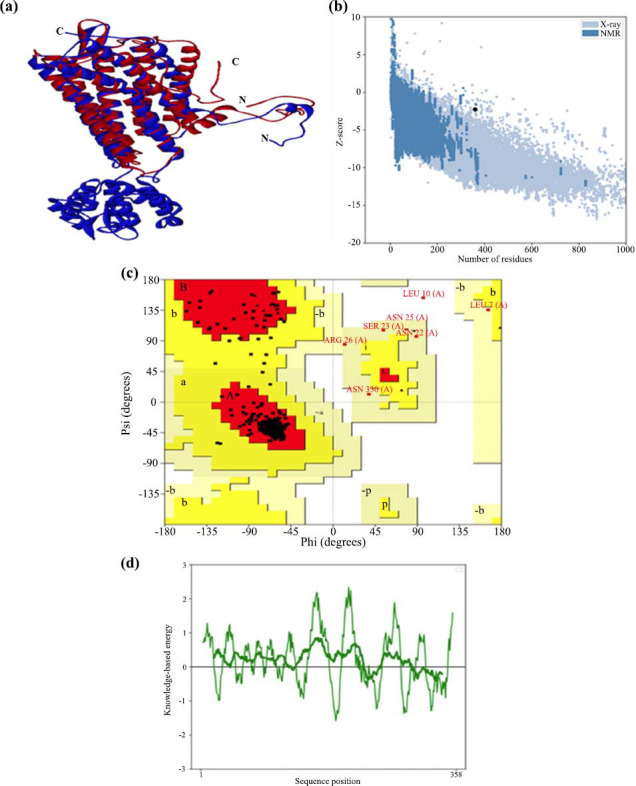
(**a**) Ribbon representation and superposition of the human GPR87 protein (shown in red) and the crystal structure of the purinergic receptor P2Y12R in complex with 2MeSADP and Gi (PDB:7XXI_A) as a template (shown in blue). The coils represent helices, the flattened parts represent β-sheets, and the remaining regions represent loops, with the N-terminus to the C-terminus. The figure was prepared using PyMOL software. (**b**) ProSA plot of the z-score, which shows the overall model quality. (**c**) Ramachandran plot. (**d**) Local model quality plot, which shows energies as a function of amino acid sequence position in the modeled 3D structure of the GPR87 protein.

The validation of the modeled protein using the ProSA web server yielded a Z-score of -2.26 (Fig. **1b**), which falls within the range typically observed for native crystal structures. Additionally, the ProSA plot shows that nearly all amino acid residues have negative energy values, further confirming that the predicted model is reliable and acceptable. The Verify-3D analysis showed that 85.45% of the residues in the final GPR87 model had an average 3D-1D score greater than 0.09, confirming the reliability of the 3D structure. Additionally, the geometric environment profile, calculated using the ERRAT tool, yielded a score of 97.86, indicating high model quality (with values above 95% considered high resolution). The Procheck V3.0 structure validation server returned an overall G-factor of -0.37, with dihedral G-factors of -0.18 and covalent G-factors of -0.74, indicating that the quality of the modeled 3D structure was within acceptable limits. Fig. (**1c**) shows that 93.7% of residues have phi-psi angles in the most preferred regions, 4.2% in additional allowed regions, 1.8% in generously allowed regions, and the remaining 0.3% in banned regions. To enhance the model's reliability, the prohibited residues were addressed using the structural refinement techniques in DSv2.5, which included loop modeling, side chain refinement, and energy minimization. The conserved sequence was analyzed using multiple sequence alignment with the template (PDB ID: 7XXI|A), revealing 131 out of 358 conserved residues. The GPR87 model was further validated by DOPE scores using the DSv2.5 (Profile3D module), with the AlphaFold2 model achieving a high DOPE score of -46854.225288. The structural properties of the modeled GPR87 were compared to those of the purinergic receptor P2Y12R (template) bound to 2MeSADP and Gi, using various validation tools, such as PROCHECK, Verify-3D, and ERRAT plots. This confirmed the stereochemical accuracy of the energy-minimized GPR87 model (Fig. **1d**).

The secondary structure of GPR87 consisted of 11 α-helices, including 7 transmembrane helices (7TMs) and 4 small 3_10_ α-helices, along with two antiparallel β-sheets. The structure was divided into two regions: intracellular and extracellular, as shown in Fig. (**2**). The overall secondary structure composition of GPR87 was as follows: 39.1% helix, 20.7% strand, and 40.2% loop. Functional analysis of the GPR87 protein revealed five N-glycosylation sites, which were crucial for the post-translational process involving the transfer of an oligosaccharide chain to an asparagine residue. These sites were located at ^4^NLTL^7^, ^25^NRSD^28^, ^33^NTTL^36^, ^72^NKTS^75^, and ^251^NQSI^254^. Additionally, there were two phosphorylation sites for tyrosine kinase and casein kinase II at ^143^KPF-Y^146^, ^345^RRS-Y^352^, ^278^SHLD^281^, and ^333^TRSE^336^, respectively.

Moreover, six protein kinase C phosphorylation sites were identified at ^236^SSR^238^, ^244^SSR^246^, ^253^SIR^255^, ^322^SRR^324^, ^337^SIR^339^, and ^343^SVR^345^. GPR87 contains a single N-myristoylation site at residues 224-229, which is crucial for anchoring the G protein to the inner surface of the plasma membrane. Signal transduction *via* GPR87 involved several post-translational modifications, including palmitoylation of the α subunit, prenylation of the γ subunit, and myristoylation, all of which are essential for proper localization and interaction of the G protein with its receptor [69].

In addition to these, certain undiscovered and hidden functions of GPR87 were predicted using the machine learning approach of Support Vector Machine (SVM). These functions were assigned to the following functional families: transmembrane (99.2%), 7 transmembrane receptors (rhodopsin family and chemoreceptor, 99.1%), and zinc-binding (94.7%). The analysis of glycoprotein M (matrix protein) by SVMProt suggests that it is a transmembrane region protein that participates in the Type II (general) secretory pathway (IISP) (58.6%) and other activities. It has previously been reported that in the human GPR87 protein, there is a sequence conflict at the position of Ser154, which is of unknown origin. This conflict may arise from sequencing errors or could represent an uncharacterized natural polymorphism (SNP) in UniProtKB [70].

Additionally, a crucial DRY motif (Asp-Arg-Tyr sequence), located at the end of transmembrane helix 3 (residues 138-140), plays a key role in G-protein interaction and signal transduction activation [71]. The DRY motif of GPR87 is positioned at the beginning of the second intracellular loop, a critical region for discovering novel lead compounds that can be designed based on ligand-protein interaction studies. Similarly, the template (PDB ID: 7XXI_A) contains a DRY motif (^123^DRY^125^) involved in G-protein activation and signal transduction [22]. To summarize, the C-terminus of GPR87 likely provides the driving force for stable interactions between the receptor, β-arrestin, and the DRY motif, playing a significant role in β-arrestin binding and maintaining receptor conformation.

### 3.3 Transmembrane Helices Analysis

Different locations of the extracellular N-terminal domain and transmembrane domain of GPR87 were previously published using 12 servers [56]. In this study, however, we utilized 13 transmembrane prediction servers, including PSI-PRED, and compared the predicted template (PDBID: 7XXI_A). The servers used included DAS, TMHMM2.0, HMMTOP, TMpred, TopPred, SOSUI, SPLIT, PredictProtein (PHD), MEMSAT, Wave-TM, HMM-TM, SMART, and Philius, which are employed to predict the position and number of transmembrane regions in GPR87 protein, as summarized in Table **1**. As shown in Table **2**, the transmembrane domain of orphan GPR87 consists of six or seven transmembrane helices (TMs).

As a result of the relatively long extracellular loops in the transmembrane domain of GPR87, its 3D structure was reconstructed using suitable templates (7XXI_A). Comparative prediction analysis of the transmembrane helices indicated that the lengths of the helices ranged from 25 to 38 amino acids. Specifically, the first transmembrane helix (TM1) ranges from residues 42 to 71 (29 amino acids), TM2 ranges from residues 74 to 99 (25 amino acids), TM3 ranges from residues 111 to 149 (38 amino acids), TM4 ranges from residues 155 to 180 (25 amino acids), TM5 ranges from residues 206 to 232 (26 amino acids), TM6 ranges from residues 252 to 278 (26 amino acids), and TM7 ranges from residues 292 to 322 (30 amino acids). Notably, only six transmembrane helices were predicted by the TopPred and SOSUI servers, placing these servers in second-tier accuracy. One transmembrane helix at the sixth position of GPR87 was not identified by these servers. In comparison, the template structure also contained seven transmembrane helices, with their predicted residues ranging between 22 to 24, as detailed in Table **2**.

### 3.4 Prediction of Active Sites

The potential binding sites in the orphan GPCR87 protein of humans were characterized using various servers, including MetaPocket, LIGSITEs, PASS, Q-Site Finder, SURFNET, and CASTp. The frequently involved amino acid residues forming the binding cavities of the GPR87 protein were Lys36, Asn37, Leu45, Cys49, Tyr58, Ala64, Ile55, Leu62, His69, Lys81, Phe94, Gly105, Cys114, Arg115, Phe121, Gly148, Thr158, Ser173, Thr183, Gly200, Val201, Lys204, Val210, Arg245, Phe261, Leu281, Tyr292, Lys296, Phe301, Phe325, Arg329, Lys337, Arg345, Tyr352, and Val358. Similarly, various predicted binding pockets through the I-TASSER tool included Lys81, Arg115, Lys296, Phe325, and Val358, which were also common in the previously predicted results.

All these key residues of the GPR87 protein play a significant role in protein-ligand interaction studies. Structural, functional, and proteomic analyses of the human GPR87 protein have contributed to the discovery of novel therapeutics for squamous cell carcinoma in regions such as the lungs, cervix, head and neck (including the larynx, pharynx, tonsils, and tongue), urinary bladder, and placenta [12, 13], as well as in the metastasis of hepatocellular carcinoma [15].

### 3.5 Molecular Dynamics Simulations

Molecular dynamics (MD) simulation is a well-established theoretical approach for studying protein behavior, including G protein-coupled receptors (GPCRs). Recent advancements have introduced target-specific scoring functions designed to identify MD snapshots that maintain the key geometric features specific to GPCRs, thus reducing the likelihood of unphysical decoys [72]. In this study, we employed the GROMACS v4.2 program to perform extended MD simulations of the GPR87 model. The simulation was conducted on a lipid bilayer within an explicit solvent environment to refine the 3D structure of GPR87. The lipid bilayer, consisting of two layers of lipid molecules, forms a thin polar membrane that mimics the cell membrane's continuous barrier. This setup provides a more realistic environment for studying the protein’s interactions and stability.

This step is crucial for ensuring the convergence of free MD. Fig. (**3A**) presents snapshots of the MD simulations over a 10 ns period, showing the Root Mean Square Deviation (RMSD) of atomic positions. Fig. (**3B**) depicts the radius of gyration (Rg). The initial jump in RMSD at 0 ps reflects the minor adjustments of the modeled protein as it equilibrates in the solvent. Throughout the simulation, the RMSD of the proposed model ranged from 0.10 to 0.50 nm, demonstrating typical behavior under the applied force field conditions. During the 1.5 ns simulation period, there was a notable increase in RMSD from the start until approximately 5 ns. By the end of the simulation, the RMSD stabilized at 0.45 nm, indicating that the molecular system reached stability. The graph shows minimal variations in RMSD thereafter, confirming that the system behaved consistently throughout the simulation. All validation results support the suitability of the GPR87 protein model for further investigation. The WHATIF server results fell within both satisfactory and acceptable ranges. Additionally, the Molprobity server indicated that 0% of the residues had poor bonds or angles, underscoring the validity of the GPR87 protein model's 3D structure.

### 3.6 Docking and MD Simulation with Lysophosphatidic Acid (LPA) Analogues

The orphan GPR87 has been deorphanized and identified as a lysophosphatidic acid receptor, which evolved from a common ancestor of the P2Y receptors. Previous studies have demonstrated that LPA activates GPR87, confirming that it functions as an LPA receptor rather than a nucleotide receptor [6]. We screened a total of 2,605 distinct LPA analogues against the target protein using blind docking with FlexX software. Out of these, 254 analogues successfully docked with the GPR87 protein, demonstrating interactions based on binding affinity. The top ten LPA analogues with the highest binding affinities to the GPR87 protein are presented in Table **3**. Lysophosphatidic acid (PubChem ID: CID:5311263) is listed at the top of Table **3**, followed by ten analogues. One of the analogues, C21H41O7P, also known as 1-Oleoyl Lysophosphatidic acid, has a molecular weight of 436.5 g/mol. In Table **3**, the basic structure of lysophosphatidic acid shows a docking score of 13.4865 with GPR87, forming two hydrogen bonds. The implicated residues in the interaction between the target protein and lysophosphatidic acid (LPA) analogues were Ser75 and Ile155, with hydrogen bond lengths of 4.70Å and 2.14Å, respectively. The docking interaction between GPR87 and LPA analogues showed binding affinities ranging from 34.0348 kJ/mol for CID:45479431 to 22.4234 kJ/mol for CID:10417707. Compared to LPA itself, the molecular docking data indicated a higher binding affinity and the formation of five hydrogen bonds with CID:45479431. The receptor residues involved in this interaction included Ser75 at TM2 (which forms three hydrogen bonds) and Ile155 (which forms two hydrogen bonds). The hydrogen bond lengths involved in the interaction between GPR87 and CID:45479431 were 4.33Å, 4.65Å, 2.28Å, 2.74Å, and 0.06Å, respectively. The α-phosphoric group of CID:45479431 interacted specifically with the TM2 and TM4 regions of GPR87. Fig. (**4**) provides a GRASP model surface depiction of this interaction, with the GPR87 protein shown in cyan. The α-phosphoric group of CID:45479431 interacted with residues in TM2 and TM4, which are highlighted within a box. Red-colored residues indicate those involved in hydrogen bonding, while black-colored residues mark the active site. The highest docking score observed for the 3D structure of GPR87 with LPA analogues, particularly CID:45479431, was 34.0348. A total of five hydrogen bonds (H-bonds) were formed, involving the residues Thr74, Ser75, Ile155, and Thr156. The side chains of the amino acids contributing to hydrogen bond formation are represented as a stick model, highlighted in green, with the residue names and numbers shown next to them. Black dotted lines represent the hydrogen bonds. These images were generated using PyMOL v0.99.

Table **4** shows snapshots of LPA and the ten LPA analogues with the highest docking scores, along with GPR87. The residues Ser75 and Ile155 are involved in the five hydrogen bonds (H-bonds) observed in the snapshot of ligand CID:45479431 with the target. The active site residues are Lys159 and Phe78.

The MD simulation of both the apo-form and the complex of GPR87 with its ligand CID: 45479431 is depicted in the graphical abstract. Throughout the 10 ns simulations, key metrics, such as the Root Mean Square Fluctuation (RMSF) of Cα-atoms, the radius of gyration, and the Root Mean Square Deviation (RMSD) of backbone atoms were monitored to assess structural changes and convergence. The target protein, GPR87, consists of 358 residues and 2925 atoms, while the ligand CID: 45479431 contains 125 atoms. As observed in the graphical abstract, at 0 ns, the protein and ligand were distant from each other, highlighting the initial separation. Before commencing MD simulations, the pKa (-6.2216205e+06) was calculated to determine if any receptor residues would adopt nonstandard ionization states. The T-coupling group in the system had 696,759 degrees of freedom at rest.

The MD simulation was conducted using GROMACSv4.2 in a POPC lipid bilayer with 113,150 water molecules under constant particle number, pressure, and temperature (NPT) with periodic boundary conditions. The system contained 20 Cl- ions and no Na+ ions, with a pressure of 0.138942 bar and a density of 1002.72 g/L. The RMSD stabilized at 0.4 nm for the apo-form and 0.3 nm for the CID: 45479431-bound GPR87 complex around 4.0 ns, as shown in the graphical abstract, indicating stable molecular behavior. Furthermore, the Radius of Gyration (Rg) value at 10 ns was recorded as 2.88911, confirming the compactness of the protein-ligand complex during the simulation. The MD simulation results for the GPR87 protein complex indicated a total free energy of -4,482,123.5 kJ/mol. The Solvent-Accessible Surface Area (SASA) was 248.415 nm^2^, and there were 1,729 hydrogen bonds formed within 0.35 nm. These values suggest a stable protein-ligand complex with extensive solvent interactions and hydrogen bonding. These findings suggest that by 4 ns, the GPR87-ligand complex adopted a relatively stable conformation compared to the apo-form, as observed in the graphical abstract. After a long MD simulation at 10 ns, the complex structure exhibited a lower RMSD value of 0.3 nm, compared to 0.45 nm for the apo-form. This implies that the ligand-docked protein became more stable during the simulation.

This study presents a comprehensive computational approach to identify potential inhibitors of the GPR87 receptor, a promising target for treating squamous cell carcinomas and adenocarcinomas. Using advanced protein modeling techniques, including Phyre2, SWISS-MODEL, I-TASSER, and AlphaFold2, we constructed and validated the structure of the human GPR87 receptor. A library of 2,605 LPA analogues was screened through *in-silico* docking, leading to the identification of high-affinity, selective candidates. Molecular dynamics simulations provided insights into the stability and behavior of the LPA-GPR87 complex. However, the use of a homology model for GPR87 comes with certain limitations. Due to the absence of a high-resolution crystal structure, the quality of the model is dependent on the templates used, and regions with low template similarity may be inaccurately represented.

Additionally, as homology models are static, they may not fully capture the receptor's dynamic conformational changes. Despite the use of validation tools like Verify-3D and ProSA, subtle inaccuracies, especially in low-resolution areas, may persist. Furthermore, the model's functional accuracy is limited due to the lack of direct experimental data on GPR87. These limitations highlight the need for caution in interpreting the results and underscore the importance of further refinement with experimental data. Nonetheless, these findings offer valuable insights for the design of targeted therapies for GPR87-related cancers.

While the computational findings presented in this study are promising, we acknowledge that experimental validation is essential to confirm the binding affinities and biological relevance of the identified inhibitors. The results from docking studies and MD simulations, though highly informative, are theoretical and require further validation through laboratory-based experiments. In particular, binding assays would be valuable for verifying the interaction between the LPA analogues and the GPR87 receptor, while cancer cell proliferation assays could provide insights into the biological effects of these inhibitors on tumor growth and survival. The current study serves as a foundation for future experimental work, and we propose that *in vitro* and *in vivo* validation be pursued as the next step in this research to assess the therapeutic potential of these inhibitors in a biological context. Such experiments would be essential for determining the true efficacy of the identified compounds and their potential for development as GPR87-targeted therapies in cancer.

## CONCLUSION

This study is the first to thoroughly explore the structural properties of the orphan GPR87 protein, identifying it as a potential therapeutic target. By examining the structural characteristics and the predicted motifs of the active site, we have gained a deeper understanding of the target protein's function and structure. Among the screened LPA analogues, CID:45479431 (FlexX score: 34.0348 kJ/mol) demonstrated superior hydrophobic interactions compared to other LPA compounds, suggesting its potential as an anti-cancer agent. Given its higher binding affinity compared to LPA, CID:45479431 should be validated for anti-cancer efficacy in both *in vivo* and *in vitro* studies. Our findings reveal that CID:45479431, a counterpart of LPA, exhibits a higher binding affinity than LPA. Consequently, we propose the use of CID:45479431 as a synergistic compound in the treatment of squamous cell carcinoma, with an emphasis on enhancing inhibitory efficiency. To achieve optimal outcomes in treating squamous cell carcinoma, high-throughput screening of diverse compounds can be conducted using the modeled GPR87 structure, predicted binding sites, and functional insights. These findings could guide the design and development of novel therapeutic agents targeting GPR87. Moreover, this study lays the groundwork for future experimental work, with *in vitro* and *in vivo* validation suggested to evaluate the therapeutic potential and efficacy of these inhibitors as GPR87-targeted cancer therapies.

## Figures and Tables

**Fig. (2):**
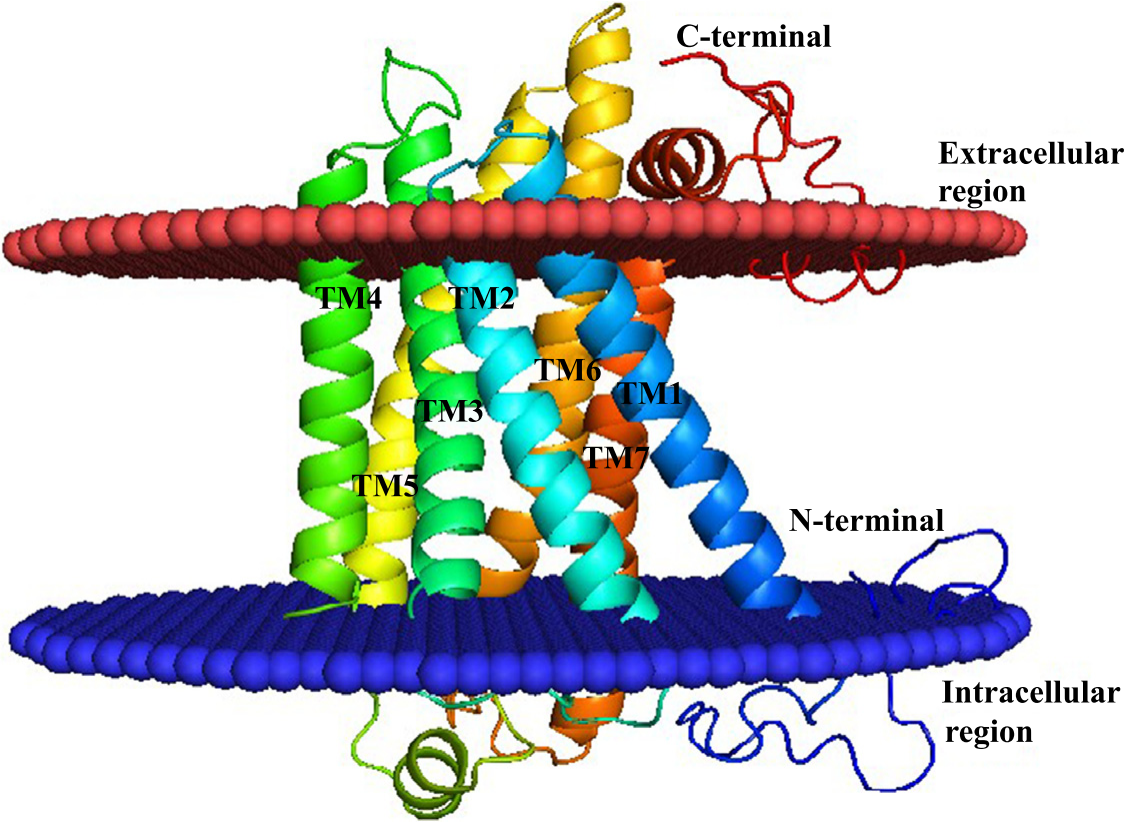
Cartoon representation of the 3D modeled structure of GPR87 protein, which shows the N-terminal in blue color and C-terminal in brown color; similarly, blue and brown plates were present, dividing protein into two regions, *i.e*., intracellular and extracellular, respectively.

**Fig. (3):**
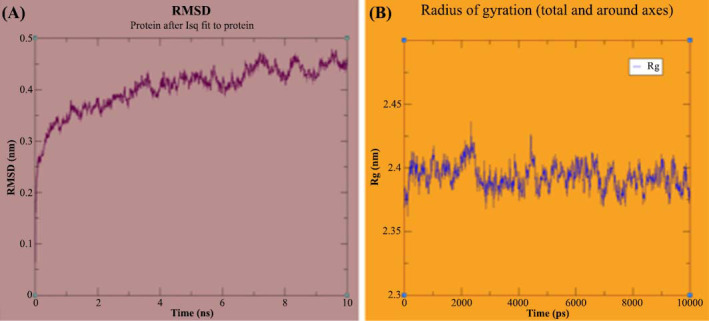
(**A**) The Root Mean Square Deviation (RMSD) of GPR87 protein obtained during 10ns of MD simulations. (**B**) It shows the Radius of Gyration of GPR87. Both graphs were plotted using the Grace tool.

**Fig. (4):**
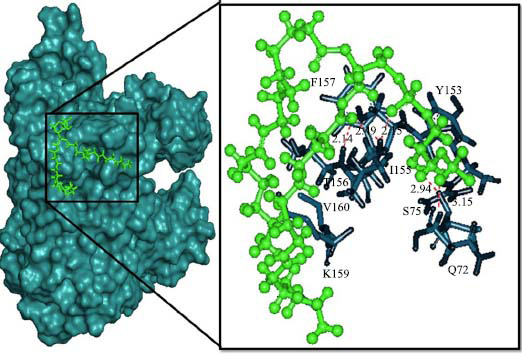
Interactions of GPR87 with lysophosphatidic acid (LPA) analogues (CID: 45479431).

**Table 1:** Summary of modeled 3D structure of GPR87 protein in human through 4 different homology modeling servers.

**Server**	**ProSA Score**	**HPM-Score**	**ERRAT Score**	**Core** **Residues**	**Allowed** **Residues**	**Generously** **Residues**	**Disallowed** **Residues**
Phyre2	-1.65	45674.10	77.38	93.4%	6.2%	0.4%	0.0%
SWISSMODEL	-1.75	17306.60	97.14	95.2%	4.4%	0.4%	0.0%
I-TASSER	-1.72	70267.50	87.10	75.8%	19.0%	2.7%	2.4%
AlphaFold2	-2.26	72276.00	97.86	93.7%	4.2%	1.8%	0.3%

**Note:** The different validation score (core residues, allowed residues, generously residues, disallowed residues) of 3D structure of GPR87 by Ramachandran plot.

**Table 2:** Predicted number and locations of transmembrane helices (TMs) in GPR87 protein in *Homo sapiens* and comparison with template (PDBID:7XXI_A).

**Servers**	**N^a^**	**TM1**	**TM2**	**TM3**	**TM4**	**TM5**	**TM6**	**TM7**
DAS	7	42-68(27)^b^	78-95(18)	111-137(27)	157-177(21)	210-229(20)	255-269(15)	300-317(18)
TMHMM	7	47-69(23)	76-98(23)	118-137(20)	157-179(23)	207-229(23)	256-278(23)	298-317(20)
HMMTOP	7	43-67(25)	74-92(19)	119-137(19)	160-179(20)	206-230(25)	253-271(19)	298-317(20)
TMpred	7	47-68(22)	76-99(24)	119-137(19)	160-178(19)	206-229(24)	253-271(19)	298-317(20)
TopPred	6	42-62(21)	-------	117-137(21)	160-180(21)	210-230(21)	252-272(21)	298-318(21)
SOSUI	6	43-65(23)	76-98(23)	127-149(23)	157-179(23)	210-232(23)	-------	296-318(23)
SPLIT	7	42- 69(28)	75-98(24)	117-138(22)	156-178(23)	208-230(23)	255-270(16)	297-319(23)
PHD	7	46-69(24)	74-95(22)	116-136(21)	158-175(18)	209-229(21)	254-271(18)	300-318(19)
MEMSAT	7	44-68(25)	76-97(22)	121-143(23)	157-178(22)	207-230(24)	250-274(25)	296-322(27)
Wave TM	7	43-71(29)	82-96(15)	111-135(25)	159-175(17)	206-229(24)	256-275(20)	300-318(19)
HMM-TM	7	47-68(22)	76-99(24)	120-137(20)	155-172(18)	207-229(23)	253-271(19)	292-314(23)
SMART	7	47-69(23)	76-98(23)	118-137(20)	157-179(23)	207-229(23)	256-278(23)	298-317(20)
Philius	7	47-68(22)	78-99(22)	118-137(20)	155-178(24)	208-230(23)	251-271(20)	298-318(21)
Template (7XXI_A)	7	27-49(22)	61-83(23)	98-120(24)	141-163(23)	193-215(23)	236-258(23)	285-304(22)

**Note:** ^a^ - Predicted number of TMs. ^b^ - Numbers in bracket are the lengths of TMs.

**Table 3:** The top ten LPA analogues with the highest binding affinity to GPR87 protein.

**S. No.**	**Lysophosphatidic Acid (LPA) Analgoues**	**FlexX Score in KJ/mol**	**No. of H-bond: Residues Involved**	**Distance of** **H-bond in Å**
1	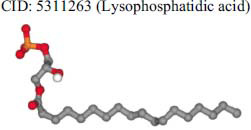	13.4865	2:Hbond Ser75H: O Ile155 H: O	4.70 2.14
2	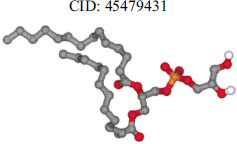	34.0348	5:Hbond Ile155:HO Ile155:HO Ser75:OH Ser75:NH Ser75:NH	2.74 0.06 4.33 4.65 2.28
3	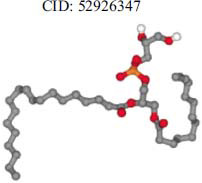	30.7981	3:Hbond Ile155: H: O Ile155: H: O Ser75:H: O	2.74 0.02 4.70
4	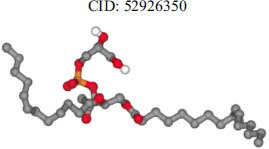	29.9800	1:Hbond Ala170:HO	1.43
5	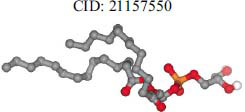	28.7233	1:Hbond Tyr116	1.83
6	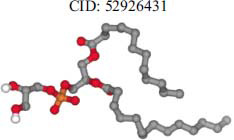	27.7332	3:Hbond Ser75:OH Ser75:OH Ser75:NH	4.70 4.35 1.74
7	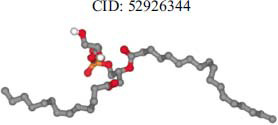	26.0131	4:Hbond Ser55:NH Ser55:OH Ser55:OH Ile155: HO	2.42 4.50 4.70 2.52
8	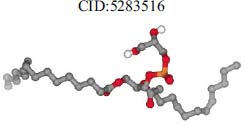	25.4660	2:Hbond Phe67:OH Lys81: NH	4.70 4.70
9	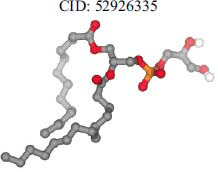	24.5242	5:Hbond Ser55:NH Ser55:OH Ser55:OH Ile155: HO Ile155: HO	2.42 4.50 4.70 2.15 1.11
10	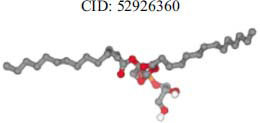	23.6416	5:Hbond Ser55:NH Ser55:OH Ser55:OH Ile155: HO	1.15 4.15 3.96 2.74
11	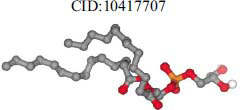	22.4234	2:Hbond Phe68:OH Phe68:OH	1.32 2.31

**Table 4:** Snapshots of LPA and the ten LPA analogues with the highest dock score with GPR87.

**S. No.**	**Ligands**	**Snapshots of LPA Analogues Interaction with GPR87**
1	CID: 5311263(Lysophosphatidic acid)	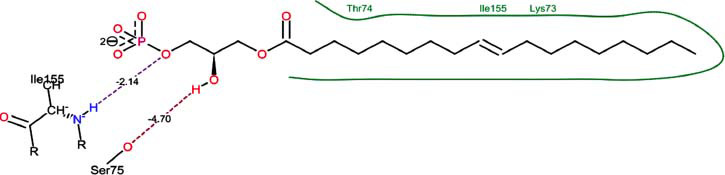
2	CID: 45479431	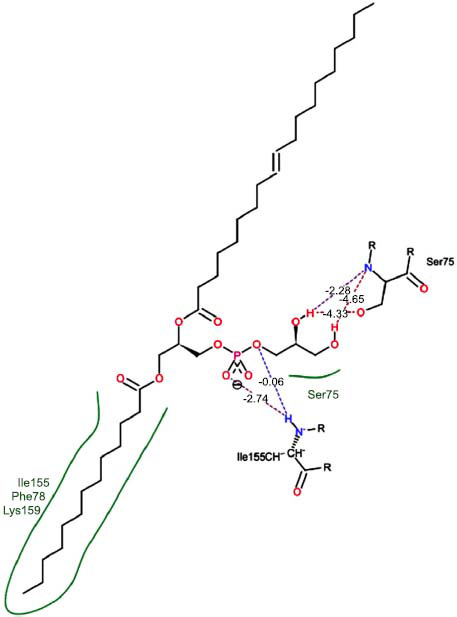
3	CID: 52926347	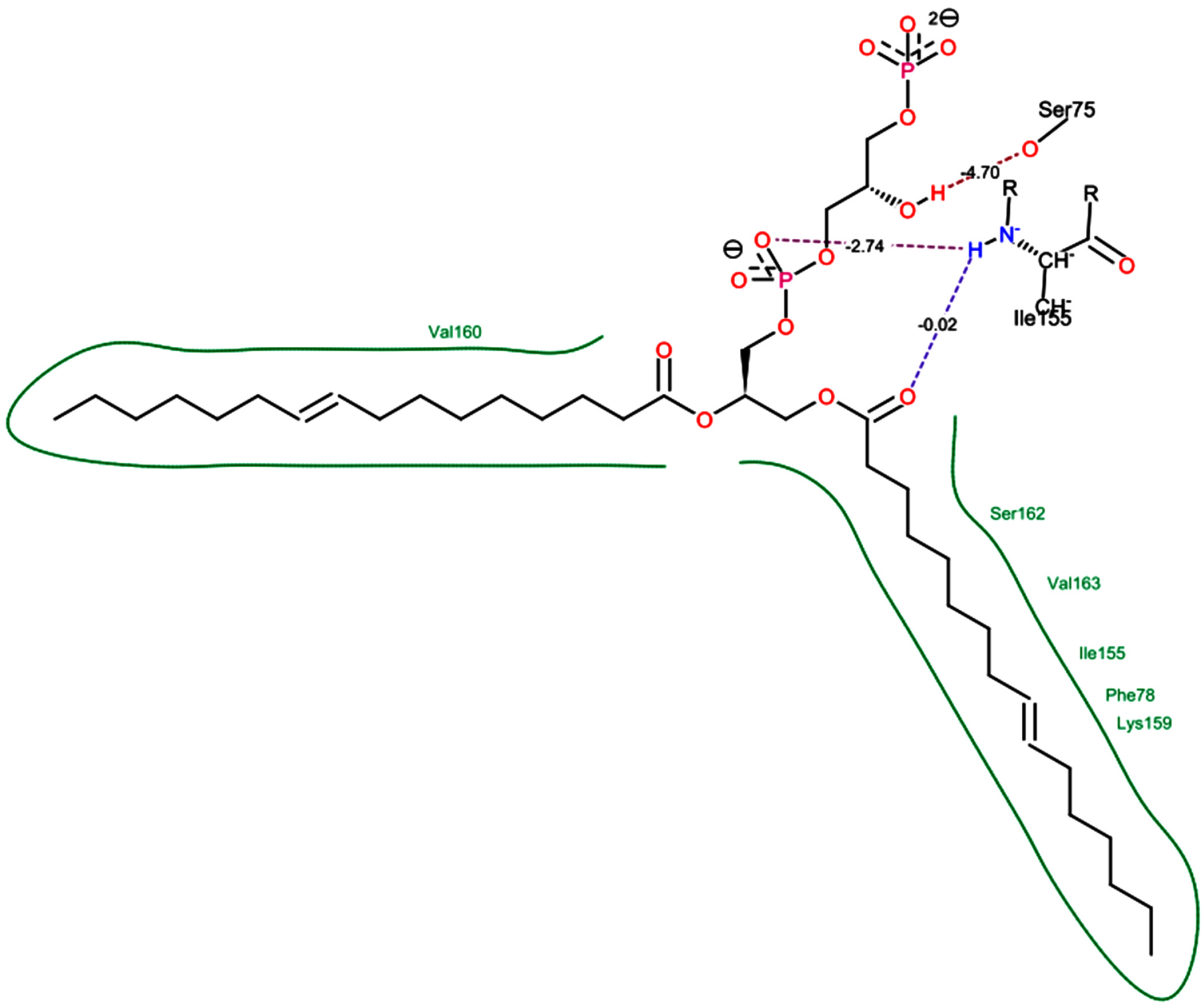
4	CID: 52926350	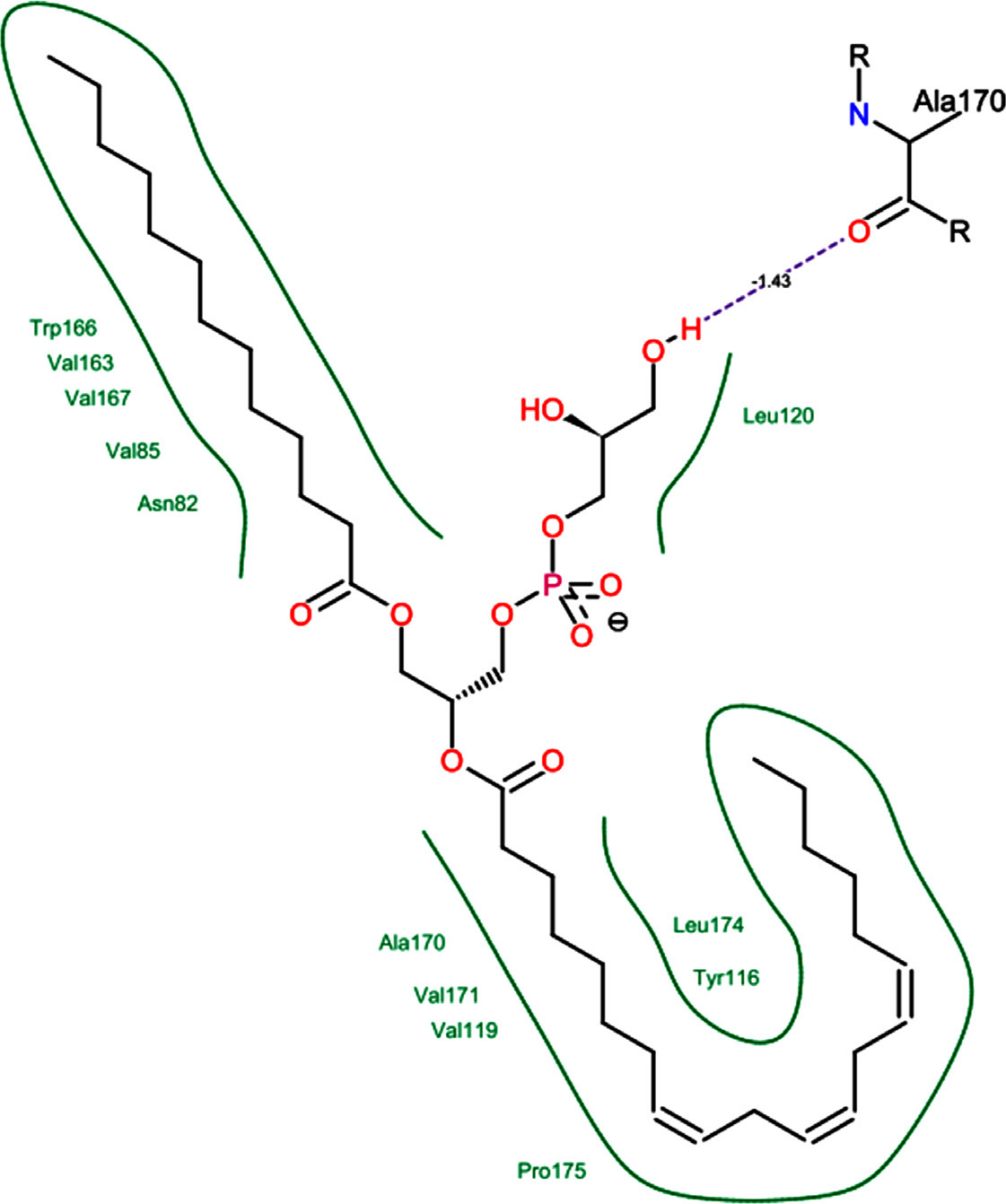
5	CID: 21157550	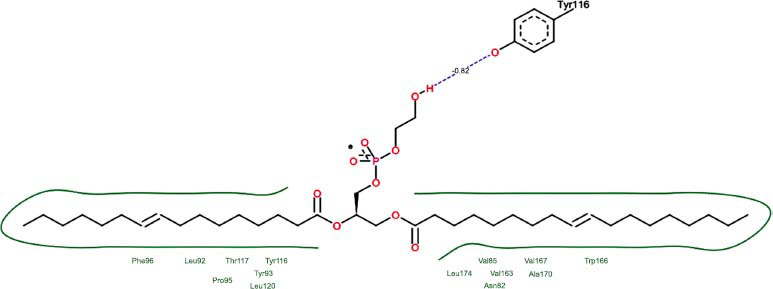
6	CID: 52926431	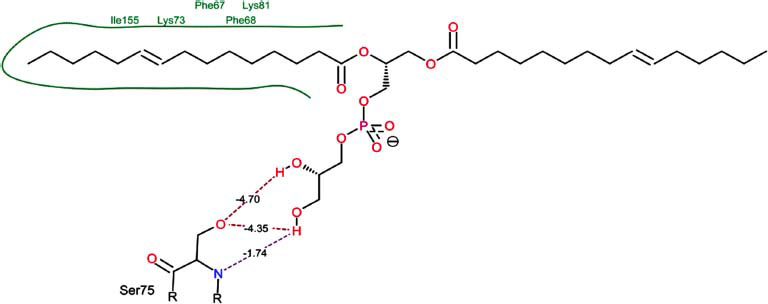
7	CID: 52926344	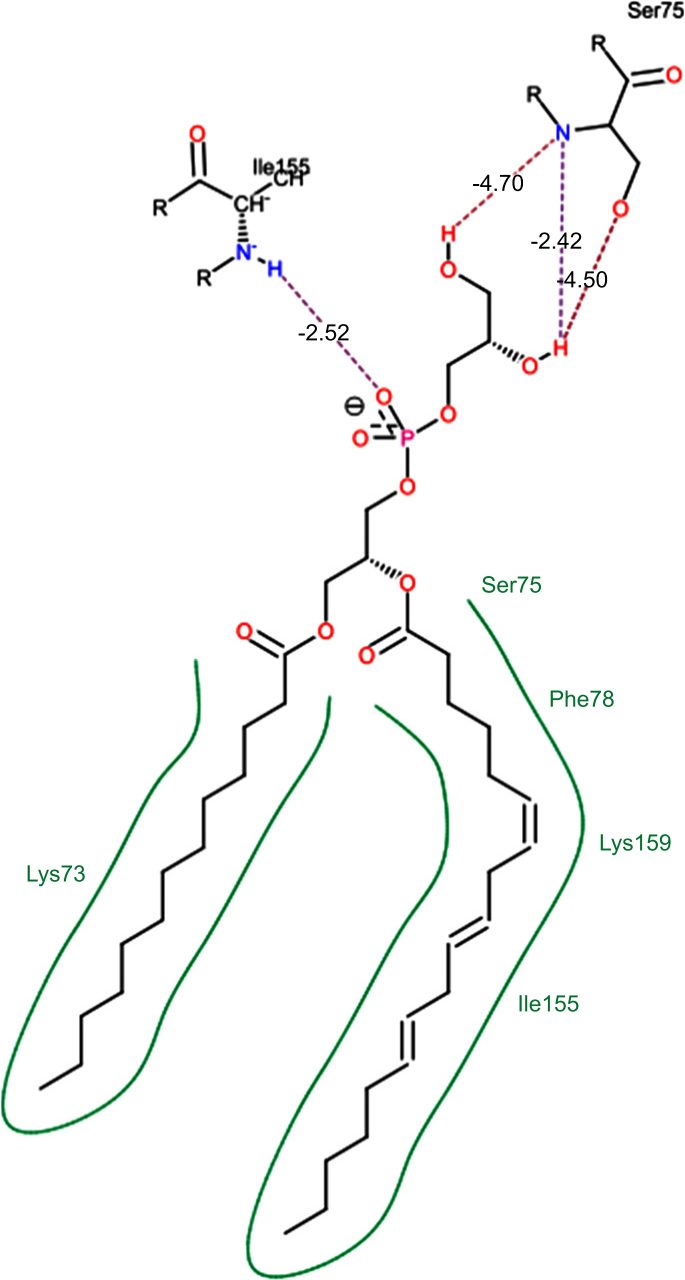
8	CID: 5283516	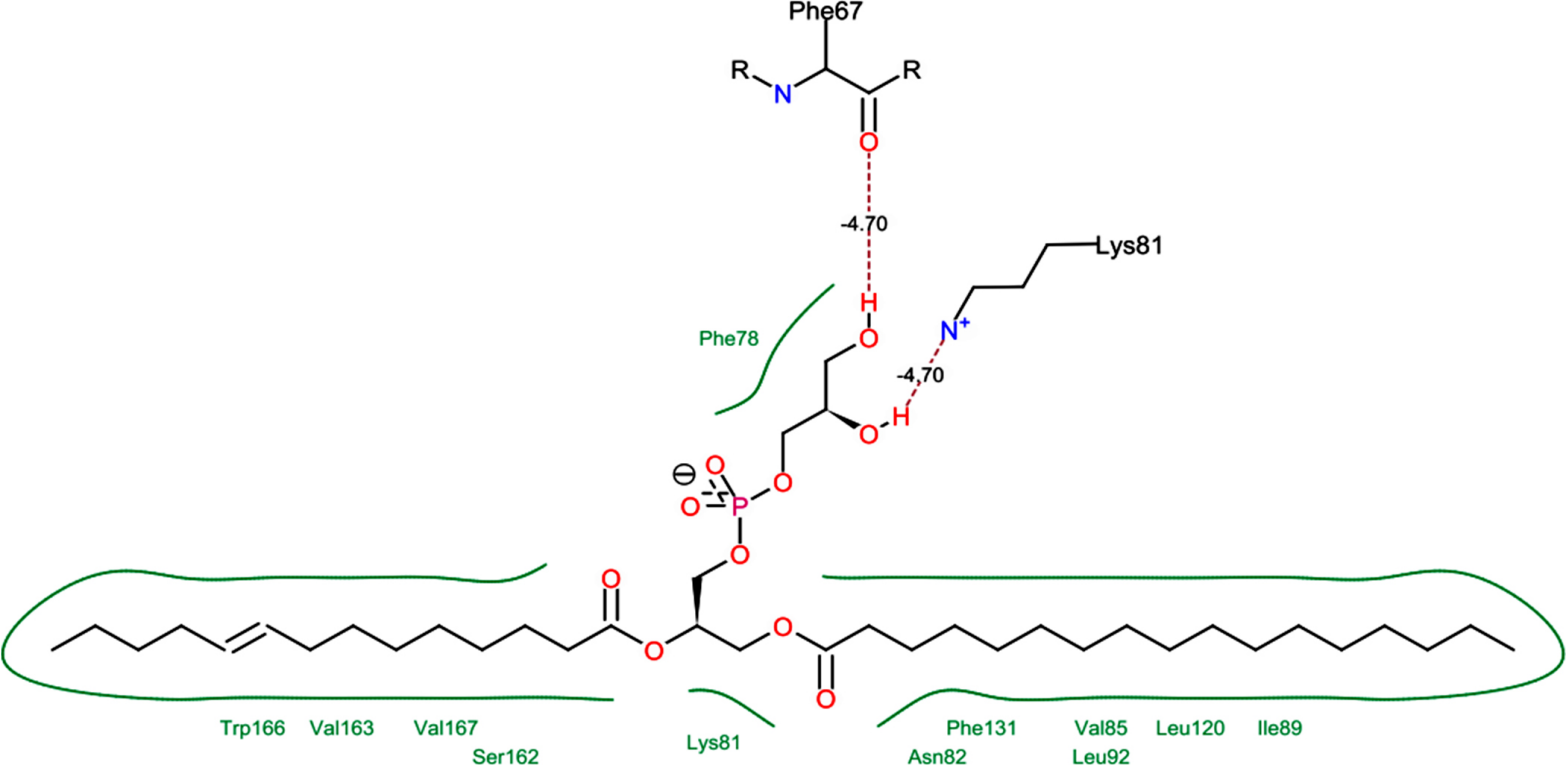
9	CID: 52926335	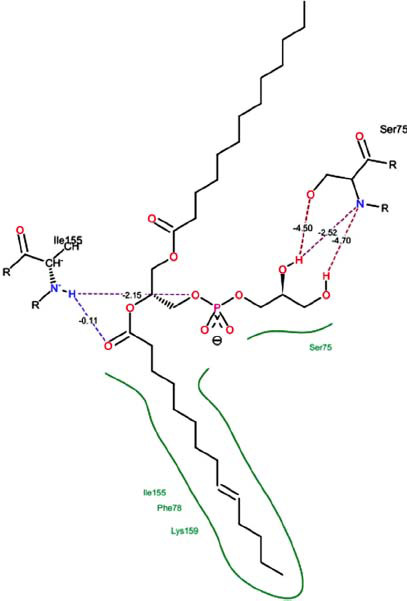
10	CID: 52926360	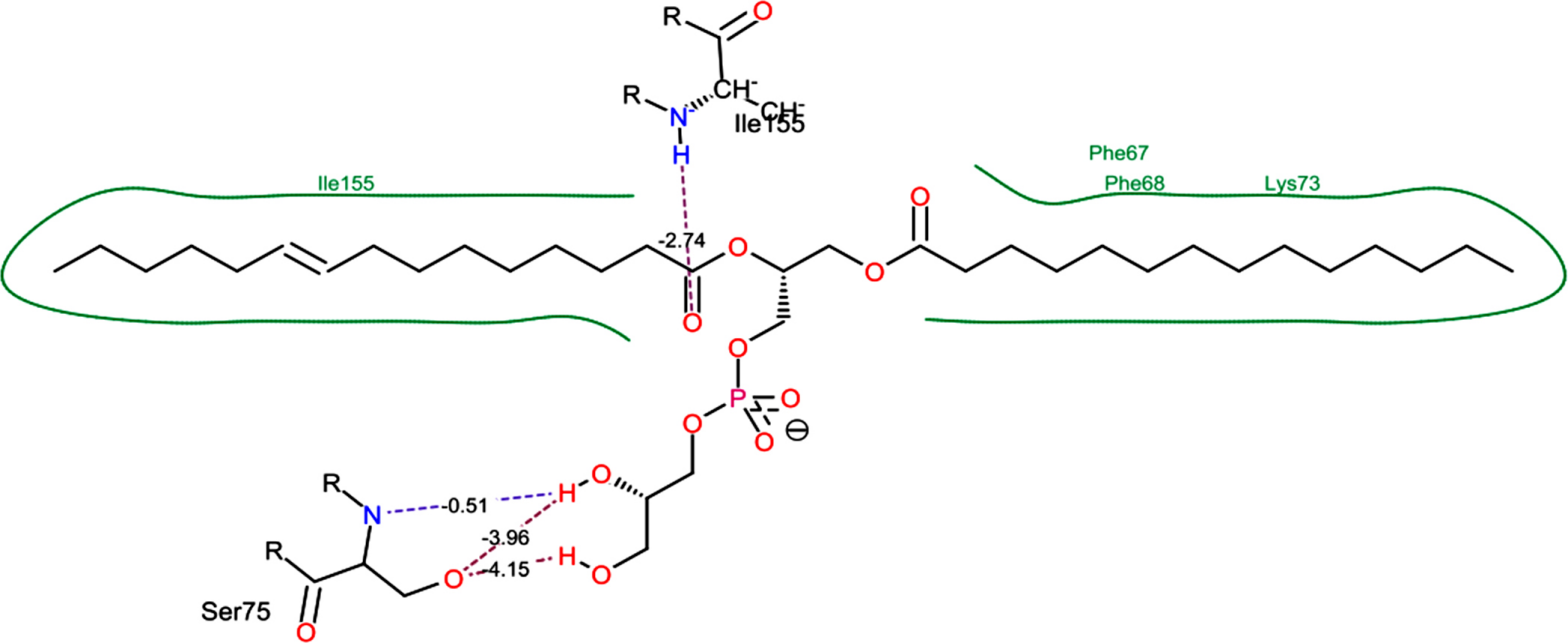
11	CID:10417707	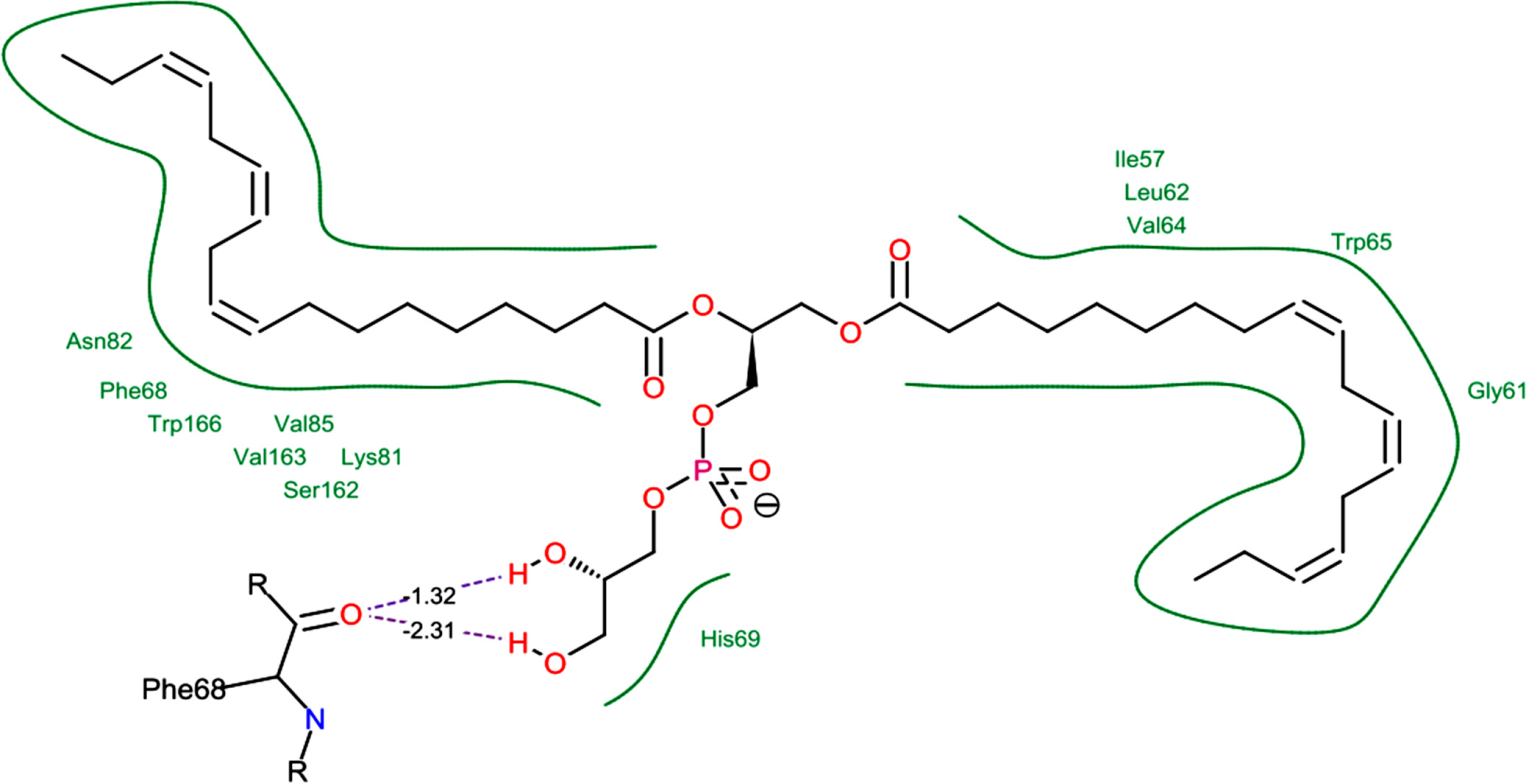

## Data Availability

The data supporting the findings of the article are available within the article.

## LIST OF ABBREVIATIONS

CKD: Chronic Kidney Disease
DOPE: Discrete Optimized Protein Energy
GPCR: G-protein Coupled Receptor
HCC: Hepatocellular Carcinoma
hGPR87: Human G-protein Coupled Receptor 87
LPA: Lysophosphatidic Acid
MD: Molecular Dynamics
NCBI: National Centre for Biotechnology Information
PDB: Protein Data Bank
PME: Particle-mesh Ewald
RMSD: Root Mean Square Deviation
SCCs: Squamous Cell Carcinomas
TMs: Transmembrane Helices

## References

[1] Civelli O. (2005). GPCR deorphanizations: The novel, the known and the unexpected transmitters.. Trends Pharmacol. Sci..

[2] Costanzi S. (2011). Homology modeling of class a G protein-coupled receptors.. Methods Mol. Biol..

[3] Bräuner-Osborne H., Wellendorph P., Jensen A. (2007). Structure, pharmacology and therapeutic prospects of family C G-protein coupled receptors.. Curr. Drug Targets.

[4] Dorsam R.T., Gutkind J.S. (2007). G-protein-coupled receptors and cancer.. Nat. Rev. Cancer.

[5] Shore D.M., Reggio P.H. (2015). The therapeutic potential of orphan GPCRs, GPR35 and GPR55.. Front. Pharmacol..

[6] Tabata K., Baba K., Shiraishi A., Ito M., Fujita N. (2007). The orphan GPCR GPR87 was deorphanized and shown to be a lysophosphatidic acid receptor.. Biochem. Biophys. Res. Commun..

[7] Chung S., Funakoshi T., Civelli O. (2008). Orphan GPCR research.. Br. J. Pharmacol..

[8] Audet M., Bouvier M. (2012). Restructuring G-protein- coupled receptor activation.. Cell.

[9] Sadiq S.K., Guixa-Gonzalez R., Dainese E., Pastor M., De Fabritiis G., Selent J. (2012). Molecular modeling and simulation of membrane lipid-mediated effects on GPCRs.. Curr. Med. Chem..

[10] Nonaka Y., Hiramoto T., Fujita N. (2005). Identification of endogenous surrogate ligands for human P2Y12 receptors by in silico and in vitro methods.. Biochem. Biophys. Res. Commun..

[11] Wittenberger T., Schaller H.C., Hellebrand S. (2001). An expressed sequence tag (EST) data mining strategy succeeding in the discovery of new G-protein coupled receptors.. J. Mol. Biol..

[12] Singh R.K. (2022). Key Heterocyclic Cores for Smart Anticancer Drug–Design Part I..

[13] Gugger M., White R., Song S., Waser B., Cescato R., Rivière P., Reubi J.C. (2008). GPR87 is an overexpressed G-protein coupled receptor in squamous cell carcinoma of the lung.. Dis. Markers.

[14] Zhang Y., Qian Y., Lu W., Chen X. (2009). The G protein-coupled receptor 87 is necessary for p53-dependent cell survival in response to genotoxic stress.. Cancer Res..

[15] Yan M., Li H., Zhu M., Zhao F., Zhang L., Chen T. (2013). G protein-coupled receptor 87 (GPR87) promotes the growth and metastasis of CD133+ cancer stem-like cells in hepatocellular carcinoma.. PLoS ONE.

[16] Kerley-Hamilton J.S., Pike A.M., Li N., DiRenzo J., Spinella M.J.A. (2005). p53-dominant transcriptional response to cisplatin in testicular germ cell tumor-derived human embyronal carcinoma.. Oncogene.

[17] Andradas C., Caffarel M.M., Pérez-Gómez E., Salazar M., Lorente M., Velasco G., Guzmán M., Sánchez C. (2011). The orphan G protein-coupled receptor GPR55 promotes cancer cell proliferation via ERK.. Oncogene.

[18] Sud N., Sharma R., Ray R., Chattopadhyay T.K., Ralhan R. (2006). Differential expression of G-protein coupled receptor 56 in human esophageal squamous cell carcinoma.. Cancer Lett..

[19] Cui X., Shi E., Li J., Li Y., Qiao Z., Wang Z., Liu M., Tang W., Sun Y., Zhang Y., Xie Y., Zhen J., Wang X., Yi F. (2022). GPR87 promotes renal tubulointerstitial fibrosis by accelerating glycolysis and mitochondrial injury.. Free Radic. Biol. Med..

[20] Ochiai S., Furuta D., Sugita K., Taniura H., Fujita N. (2013). GPR87 mediates lysophosphatidic acid-induced colony dispersal in A431 cells.. Eur. J. Pharmacol..

[21] Altschul S.F., Gish W., Miller W., Myers E.W., Lipman D.J. (1990). Basic local alignment search tool.. J. Mol. Biol..

[22] Li B., Han S., Wang M., Yu Y., Ma L., Chu X., Tan Q., Zhao Q., Wu B. (2022). Structural insights into signal transduction of the purinergic receptors P2Y1R and P2Y12R.. Protein Cell.

[23] Larkin M.A., Blackshields G., Brown N.P., Chenna R., McGettigan P.A., McWilliam H., Valentin F., Wallace I.M., Wilm A., Lopez R., Thompson J.D., Gibson T.J., Higgins D.G. (2007). Clustal W and Clustal X version 2.0.. Bioinformatics.

[24] Martí-Renom M.A., Stuart A.C., Fiser A., Sánchez R., Melo F., Šali A. (2000). Comparative protein structure modeling of genes and genomes.. Annu. Rev. Biophys. Biomol. Struct..

[25] (2003). Cerius2 Modeling Environment, Release 4.7.

[26] Yang J., Zhang Y. (2015). I-TASSER server: New development for protein structure and function predictions.. Nucleic Acids Res..

[27] Kelley L.A., Mezulis S., Yates C.M., Wass M.N., Sternberg M.J.E. (2015). The Phyre2 web portal for protein modeling, prediction and analysis.. Nat. Protoc..

[28] Waterhouse A., Bertoni M., Bienert S., Studer G., Tauriello G., Gumienny R., Heer F.T., de Beer T.A.P., Rempfer C., Bordoli L., Lepore R., Schwede T. (2018). SWISS-MODEL: Homology modelling of protein structures and complexes.. Nucleic Acids Res..

[29] Jumper J., Evans R., Pritzel A., Green T., Figurnov M., Ronneberger O., Tunyasuvunakool K., Bates R., Žídek A., Potapenko A., Bridgland A., Meyer C., Kohl S.A.A., Ballard A.J., Cowie A., Romera-Paredes B., Nikolov S., Jain R., Adler J., Back T., Petersen S., Reiman D., Clancy E., Zielinski M., Steinegger M., Pacholska M., Berghammer T., Bodenstein S., Silver D., Vinyals O., Senior A.W., Kavukcuoglu K., Kohli P., Hassabis D. (2021). Highly accurate protein structure prediction with AlphaFold.. Nature.

[30] De Lano W.L. (2002). The PyMOL molecular graphics system,. http://www.pymol.org.

[31] Laskowski R.A., MacArthur M.W., Moss D.S., Thornton J.M. (1993). PROCHECK: A program to check the stereochemical quality of protein structures.. J. Appl. Cryst..

[32] Colovos C., Yeates T.O. (1993). Verification of protein structures: Patterns of nonbonded atomic interactions.. Protein Sci..

[33] Bowie J.U., Lüthy R., Eisenberg D. (1991). A method to identify protein sequences that fold into a known three-dimensional structure.. Science.

[34] Wiederstein M., Sippl M.J. (2007). ProSA-web: Interactive web service for the recognition of errors in three-dimensional structures of proteins.. Nucleic Acids Res..

[35] Shen M., Sali A. (2006). Statistical potential for assessment and prediction of protein structures.. Protein Sci..

[36] Bryson K., McGuffin L.J., Marsden R.L., Ward J.J., Sodhi J.S., Jones D.T. (2005). Protein structure prediction servers at University College London Nucleic Acid.. Research.

[37] Cserzö M., Wallin E., Simon I., von Heijne G., Elofsson A. (1997). Prediction of transmembrane alpha-helices in prokaryotic membrane proteins: The dense alignment surface method.. Protein Eng. Des. Sel..

[38] Krogh A., Larsson B., von Heijne G., Sonnhammer E.L.L. (2001). Predicting transmembrane protein topology with a hidden markov model: Application to complete genomes11Edited by F.. Cohen. J. Mol. Biol..

[39] Tusnády G.E., Simon I. (1998). Principles governing amino acid composition of integral membrane proteins: Application to topology prediction.. J. Mol. Biol..

[40] Hofmann K., Stoffel W. (1993). TMbase: A database of membrane spanning proteins segments.. Biol. Chem. Hoppe Seyler.

[41] Claros M.G., Heijne G. (1994). TopPred II: An improved software for membrane protein structure predictions.. Bioinformatics.

[42] Hirokawa T., Boon-Chieng S., Mitaku S. (1998). SOSUI: Classification and secondary structure prediction system for membrane proteins.. Bioinformatics.

[43] Juretić D., Zoranić L., Zucić D. (2002). Basic charge clusters and predictions of membrane protein topology.. J. Chem. Inf. Comput. Sci..

[44] Rost B., Yachdav G., Liu J. (2004). The predict protein server.. Nucleic Acids Res..

[45] Jones D.T., Taylor W.R., Thornton J.M. (1994). A model recognition approach to the prediction of all-helical membrane protein structure and topology.. Biochemistry.

[46] Bagos P.G., Liakopoulos T.D., Hamodrakas S.J. (2006). Algorithms for incorporating prior topological information in HMMs: Application to transmembrane proteins.. BMC Bioinformatics.

[47] Schultz J., Milpetz F., Bork P., Ponting C.P. (1998). SMART, a simple modular architecture research tool: Identification of signaling domains.. Proc. Natl. Acad. Sci. USA.

[48] Reynolds S.M., Käll L., Riffle M.E., Bilmes J.A., Noble W.S. (2008). Transmembrane topology and signal peptide prediction using dynamic bayesian networks.. PLOS Comput. Biol..

[49] Gallwitz B., Witt M., Paetzold G., Morys-Wortmann C., Zimmermann B., Eckart K., Fölsch U.R., Schmidt W.E. (1994). Structure/activity characterization of glucagon-like peptide-1.. Eur. J. Biochem..

[50] Zhang Z., Li Y., Lin B., Schroeder M., Huang B. (2011). Identification of cavities on protein surface using multiple computational approaches for drug binding site prediction.. Bioinformatics.

[51] Huang B., Schroeder M. (2006). LIGSITEcsc: Predicting ligand binding sites using the Connolly surface and degree of conservation.. BMC Struct. Biol..

[52] Brady G.P., Stouten P.F.W. (2000). Fast prediction and visualization of protein binding pockets with PASS.. J. Comput. Aided Mol. Des..

[53] Laurie A.T.R., Jackson R.M. (2005). Q-SiteFinder: An energy-based method for the prediction of protein-ligand binding sites.. Bioinformatics.

[54] Laskowski R.A. (1995). SURFNET: A program for visualizing molecular surfaces, cavities, and intermolecular interactions.. J. Mol. Graph..

[55] Dundas J., Ouyang Z., Tseng J., Binkowski A., Turpaz Y., Liang J. (2006). CASTp: Computed atlas of surface topography of proteins with structural and topographical mapping of functionally annotated residues.. Nucleic Acids Res..

[56] Rani M., Nischal A., Sahoo G.C., Khattri S. (2013). Computational analysis of the 3-D structure of human GPR87 protein: Implications for structure-based drug design.. Asian Pac. J. Cancer Prev..

[57] Van Der Spoel D., Lindahl E., Hess B., Groenhof G., Mark A.E., Berendsen H.J.C. (2005). GROMACS: Fast, flexible, and free.. J. Comput. Chem..

[58] Berendsen H.J.C., Grigera J.R., Straatsma T.P. (1987). The missing term in effective pair potentials.. J. Phys. Chem..

[59] Humphrey W., Dalke A., Schulten K. (1996). VMD: Visual molecular dynamics.. J. Mol. Graph..

[60] Xu Y., Shen Z., Wiper D.W., Wu M., Morton R.E., Elson P., Kennedy A.W., Belinson J., Markman M., Casey G. (1998). Lysophosphatidic acid as a potential biomarker for ovarian and other gynecologic cancers.. JAMA.

[61] Abu-Dief A.M., El-Khatib R.M., El-Dabea T., Abdou A., Aljohani F.S., Al-Farraj E.S., Barnawi I.O., El-Remaily M.A.E.A.A.A. (2023). Fabrication, structural elucidation of some new metal chelates based on N-(1H-Benzoimidazol-2-yl)-guanidine ligand: DNA interaction, pharmaceutical studies and molecular docking approach.. J. Mol. Liq..

[62] Rarey M., Kramer B., Lengauer T., Klebe G. (1996). A fast flexible docking method using an incremental construction algorithm.. J. Mol. Biol..

[63] Rarey M., Kramer B., Lengauer T. (1999). The particle concept: Placing discrete water molecules during protein-ligand docking predictions.. Proteins.

[64] Böhm H.J. (1992). The computer program LUDI: A new method for the de novo design of enzyme inhibitors.. J. Comput. Aided Mol. Des..

[65] Rani M., Dikhit M.R., Sahoo G.C., Das P. (2011). Comparative domain modeling of human EGF-like module EMR2 and study of interaction of the fourth domain of EGF with chondroitin 4-sulphate.. J. Biomed. Res..

[66] Sahoo G.C., Dikhit M.R., Rani M., Ansari M.Y., Jha C., Rana S., Das P. (2013). Analysis of sequence, structure of GAPDH of Leishmania donovani and its interactions.. J. Biomol. Struct. Dyn..

[67] Rani M., Sharma A.K., Chouhan R.S., Sur S., Mansuri R., Singh R.K. (2024). Natural flavonoid pectolinarin computationally targeted as a promising drug candidate against SARS-CoV-2.. Curr. Res. Struct. Biol..

[68] Téletchéa S., Esque J., Urbain A., Etchebest C., de Brevern A.G. (2023). Evaluation of transmembrane protein structural models using HPMScore.. BioMedInformatics.

[69] Wall M.A., Coleman D.E., Lee E., Iñiguez-Lluhi J.A., Posner B.A., Gilman A.G., Sprang S.R. (1995). The structure of the G protein heterotrimer Giα1β1γ2.. Cell.

[70] Ota T., Suzuki Y., Nishikawa T., Otsuki T., Sugiyama T., Irie R., Wakamatsu A. (2004). Complete sequencing and characterization of 21,243 full-length human cDNAs.. Nat. Genet..

[71] Kim K.M., Caron M.G. (2008). Complementary roles of the DRY motif and C-terminus tail of GPCRS for G protein coupling and β-arrestin interaction.. Biochem. Biophys. Res. Commun..

[72] Heifetz A., Barker O., Morris G.B., Law R.J., Slack M., Biggin P.C. (2013). Toward an understanding of agonist binding to human Orexin-1 and Orexin-2 receptors with G-protein-coupled receptor modeling and site-directed mutagenesis.. Biochemistry.

